# One-Pot Protolignin Extraction by Targeted Unlocking Lignin–Carbohydrate Esters via Nucleophilic Addition–Elimination Strategy

**DOI:** 10.34133/research.0069

**Published:** 2023-03-09

**Authors:** Yuhan Lou, Xinyue Sun, Yanyan Yu, Suqing Zeng, Yilin Li, Yongzhuang Liu, Haipeng Yu

**Affiliations:** Key Laboratory of Bio-Based Material Science and Technology of Ministry of Education, Northeast Forestry University, Harbin 150040, P. R. China.

## Abstract

Protolignin extraction can facilitate structure elucidation and valorization of lignin in biorefinery, but is rather challenging due to the complex chemical bonds present. Here, we developed the in situ generated NH_3_-reline (IGNR) system to realize one-pot protolignin extraction from lignocellulose. In the IGNR system, reline consisting of choline chloride and urea acted as both a solvent and a nucleophile generator, and the nucleophilic addition–elimination mechanism was verified by model compound studies. The in situ generated NH_3_ could precisely cleave the lignin–carbohydrate esters in lignocellulose with a near-quantitative retention of carbohydrates. The extracted IGNR–Protolignin exhibited native lignin substructure with high molecular weight and high β-O-4′ content (41.5 per 100 aromatic units). In addition, the up-scaled kilogram reaction demonstrated the feasibility of the IGNR system for potential industrial application in a green and sustainable pathway. This work represents a breakthrough toward protolignin extraction in practice with the future goal of achieving total biorefinery.

## Introduction

Lignin, along with cellulose and hemicellulose, is a major component of the plant skeleton and contains unique aromatic building blocks. It is the second-largest amount of plant-based organic matter only after cellulose and the most abundant natural aromatic polymers [1–3]. However, unlike cellulose whose structures have been well elucidated [4,5], lignin has a very complex and variable structure, albeit current technologies of advanced in situ nuclear magnetic resonance, organic synthesis, and lignin biosynthesis contributed a lot to identifying the structure of lignin. Protolignin represents natural lignin in various parts of plant cell wall or isolated lignin without (or with minimal) structure modifications. Protolignin extraction could directly enable structure elucidation, mechanism verification, as well as aromatics production during lignin valorization [6,7]. Nevertheless, there are 2 main challenges for the protolignin extraction: (a) lignin is an extremely complex reticulated polymer with random polymerization sites between monomers via carbon–carbon and carbon–oxygen bonds without a strict fixed structure [8–11]; (b) lignin is closely entangled with hemicellulose and cellulose by hydrogen and covalent bonds (e.g., lignin–carbohydrate ester and ether linkages), which can easily cause structural changes during fractionation and lignin extraction due to the large number of reactive linkages within its structure [12–16]. Therefore, efficient extraction of protolignin with natural structure is extremely challenging.

Currently, only several protolignins or protolignin derivatives have been prepared, and relevant research is still in progress. Regarding protolignin extraction, milled wood lignin (MWL) was first proposed by Björkman in 1954 [17], and is recognized as one of the most common lignin models with the closest structure to protolignin [18]. The general procedure for MWL extraction includes mild mechanical ball milling and subsequent neural organic solvent extraction/purification processes. Currently, MWL is widely used for interpreting lignin structure, understanding the pathway for lignin biosynthesis, and determining the mechanism of lignin valorization [19,20]. As another protolignin, cellulolytic enzyme lignin (CEL) is extracted from lignocellulose by biologically digesting the carbohydrates and afterward purification with similar organic solvents [21–23]. The procedures for both MWL and CEL extraction are complicated and include a series of extraction and purification processes with organic solvents. Although they are representative and widely applied, key limitations (i.e., complex multistep procedures, long reaction period, high energy consumption, and low output) are clear obstacles for protolignin production in practice.

Protolignin derivatives (or native-like lignin) can also be extracted from lignocellulose using a “stabilization” strategy. For example, acidic catalysts or relative higher temperatures are applied to promote lignocellulose fractionation and lignin extraction, while stabilized molecules in the system are grafted onto the lignin, protecting it from subsequent depolymerization or the formation of active intermediates [24]. A representative procedure is an aldehyde stabilization strategy for lignin extraction with high aryl ether content, in which aldehydes react with α- and γ-hydroxy groups to inhibit the formation of active α carbocations (highly active for condensation) and the cleavage of β-O-4′ bonds [25,26]. Other stabilization strategies are generally achieved by the α-alkoxylation of lignin with alcohols or diols during the acid-catalyzed lignocellulose fractionation process. A series of ethanol-, butanol-, ethylene glycol-stabilized lignin derivatives have been successfully obtained with an abundance of β-O-4′ linkages [24,27–30]. Recently, flow-through devices have been used to dissolve and alkoxylate lignin in a flow-through acidic environment using methanol, ethanol, or formic acid [31–34]. Compared with the extraction methods of MWL and CEL, the extraction of stabilized protolignin derivatives is more efficient and can be valorized to aromatic monomers in high yield. However, these protolignin derivatives are still different from protolignin, albeit the content of β-O-4′ linkage is high. Efficient protolignin extraction with advanced strategies is still challenging, and further scientific and technological breakthroughs are urgently needed.

**Fig. 1.**
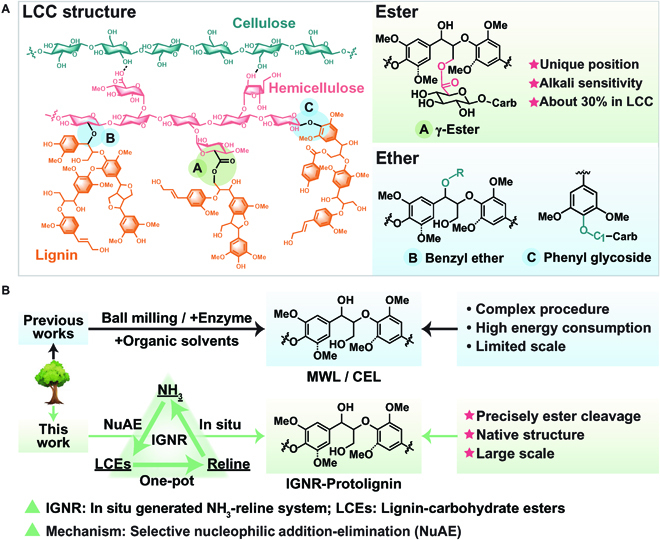
Structure–activity relationships of LCCs and methodologies for protolignin extraction. (A) Structure–activity relationships of LCCs in terms of poplar, the main ester, and ether linkages at the molecular interfaces between lignin and hemicellulose, wherein the lignin–carbohydrate ester shows the unique characteristics of single-linkage position and accounts for 30% content of total chemical linkages. (B) Methodologies for protolignin extraction in previous works and one-pot protolignin extraction based on the IGNR system in this work. The IGNR process is achieved via a selective nucleophilic addition–elimination mechanism. Ammonia gas (NH_3_) is generated by partially sacrificing urea in reline and acts as a nucleophilic reagent to attack the carbonyl carbon atom in lignin–carbohydrate esters, enabling IGNR–Protolignin extraction after the elimination process. The advantages of this work are its efficient one-pot procedure, high yield of protolignin with native structure, and potential large-scale extraction.

To solve the above problems, we summarize the structure–activity relationships of the well-known lignin–carbohydrate complexes (LCCs) at the molecular level (Fig. 1A). Lignin is mainly covalently connected with hemicellulose via phenyl ester (γ-Ester), benzyl ether (BE), and phenyl glycoside (PhGly) [35–37]. These ether and ester covalent bonds are the keys for lignocellulose fractionation and lignin extraction. Most of the current chemical mechanisms for lignin extraction are achieved by the acid-catalyzed hydrolysis or solvolysis of LCCs [38–41]. However, the cleavage of ether bonds in lignin can hardly be avoided under the above conditions, which makes the protolignin extraction extremely difficult. It was found that the ester bonds account for about 30% chemical linkages of total LCCs (typical in poplar LCCs and as calculated from literature) [12,13,15] and are almost entirely located at the molecular interfaces between lignin and hemicellulose. At the same time, the chemical reactivity of ester bonds (γ-Esters) differs from that of ether bonds (BEs and PhGlys) and is more sensitive to alkaline conditions. Therefore, inspired by the above elucidation of LCCs, it is possible to extract protolignin by selectively cleaving lignin–carbohydrate esters in LCCs.

Here, we developed a strategy for one-pot protolignin extraction from lignocellulose. A green and low-cost in situ generated NH_3_-reline (IGNR) system was developed for efficient protolignin extraction (Fig. 1B). It should be noted that reline in the IGNR system is also widely known as a typical deep eutectic solvent of choline chloride and urea [42,43]. Reline means a tractable ionic mixture with facile preparation from cheap and readily available precursors, making itself a devisable, biodegradable, and nontoxic reaction system [42]. The lignin–carbohydrate esters in the LCCs are precisely dissociated via a nucleophilic addition–elimination mechanism. Ammonia gas (NH_3_) that generated by partially sacrificing urea acts as a nucleophilic reagent to attack the carbonyl carbon atom in the lignin–carbohydrate esters. By precisely cleaving the lignin–carbohydrate esters through IGNR, the liberated IGNR–Protolignin is subsequently solubilized in the solvent and can be readily isolated, simultaneously providing the carbohydrates as an insoluble residue in nearly quantitative yield. In this study, the relevant trial was first carried out by model compound studies to verify this hypothesis. Then, the chemical structure and molecular weight of IGNR–Protolignin were thoroughly investigated and compared with MWL (as a typical protolignin). Finally, the reaction was up-scaled 100-fold to reveal the potential industrial application of this strategy. Further prospects for entirely closed-loop lignocellulose refinery based on the system were also discussed, including solvent recovery and reuse, and by-product immobilization as fertilizer. Overall, this work demonstrated a new route for protolignin extraction and promoted the development of future all-component biorefinery.

## Results

### Conception of IGNR system

**Fig. 2.**
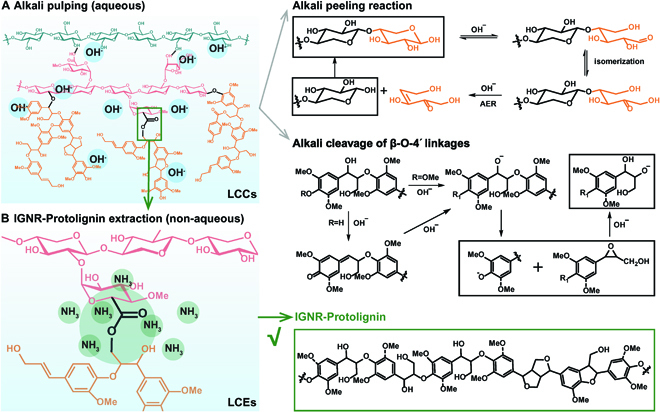
Design and reaction pathways for protolignin extraction. (A) In conventional alkali pulping systems, the alkoxy group of hemicellulose undergoes elimination decomposition (i.e., alkali peeling reaction), and the alkali cleavage of the phenolic or nonphenolic β-O-4′ occurred simultaneously. (B) In the IGNR–Protolignin extraction system, NH_3_ generated at 150 °C selectively cleaves the lignin–carbohydrate esters (LCEs) with minimal effect on either hemicellulose or lignin to eventually obtain IGNR–Protolignin with native structure.

LCC cleavage is important for lignin extraction, from either lignin-first or carbohydrate-first pathways. Acidic conditions are generally effective for LCC cleavage and lignin extraction according to previous studies [38,39]; however, lignin depolymerization or modification can hardly be avoided. Based on the consideration that lignin–carbohydrate esters are unique in LCCs and more sensitive to alkali conditions [15], it is assumed that alkali conditions will possibly provide a chance for selective cleavage of lignin–carbohydrate esters and protolignin extraction. The most common reaction for the dissociation of esters is saponification (i.e., the alkaline hydrolysis of ester bonds) in alkali pulping, which likely occurs at 140 to 170 °C. However, OH^−^ in high-temperature alkaline aqueous solutions has a hydrolytic effect on hemicellulose as well as the ability to nucleophilically cleave (non-)phenolic β-O-4′ bonds in the lignin structure (Fig. 2A, Alkali peeling reaction and alkali cleavage of β-O-4′ linkages) [44,45]. For protolignin extraction, the undesired hydrolytic depolymerization pathways of lignin and hemicellulose should be inhibited under the similar conditions. Therefore, our approach for the protolignin extraction system aims to keep the ability for cleavage of lignin–carbohydrate esters and inhibit the undesired hydrolysis reactions.

We thus present an IGNR system for selective cleavage of lignin–carbohydrate esters and protolignin extraction (Fig. 2B). Typically, the non-aqueous solvent is prepared with reline (1:2 molar ratio of choline chloride and urea). Gaseous NH_3_ is in situ generated from the partially thermal sacrifice of urea. When the described system is used for lignocellulose fractionation, a 3-phase system (NH_3_-reline-lignocellulose) would form. NH_3_ acts as a nucleophilic reagent to precisely cleave the lignin–carbohydrate esters via nucleophilic addition–elimination. In this process, NH_3_ as a gas nucleophile demonstrates obvious advantage compared with other solid or liquid nucleophilic reagents in terms of accessibility to the cell wall of lignocellulose. Meanwhile, the gas nucleophile would have minimal effect on the carbohydrate decomposition compared with other aqueous or organic solvent-based systems. The liberated IGNR–Protolignin tends to dissolve in the hydrogen bond-rich reline system prior to lignin isolation.

### Mechanism verification using model compounds

To verify the feasibility of the IGNR system for targeting cleavage of lignin–carbohydrate esters, several control experiments were performed using model compounds, including 2-(2-methoxyphenoxy)-1-(4-methoxyphenyl) ethanol (1a), phenyl β-d-glucopyranoside (1b), and benzyl benzoate (1c) as a lignin aryl ether bond, lignin–carbohydrate ether bond, and lignin–carbohydrate ester bond, respectively. For a typical IGNR reaction, the as-prepared reline solvent and model compounds were mixed and sealed in an autoclave and heated to 150 °C (starting decomposition temperature of urea), which was maintained for 6 h for continuous NH_3_ generation.

**Fig. 3.**
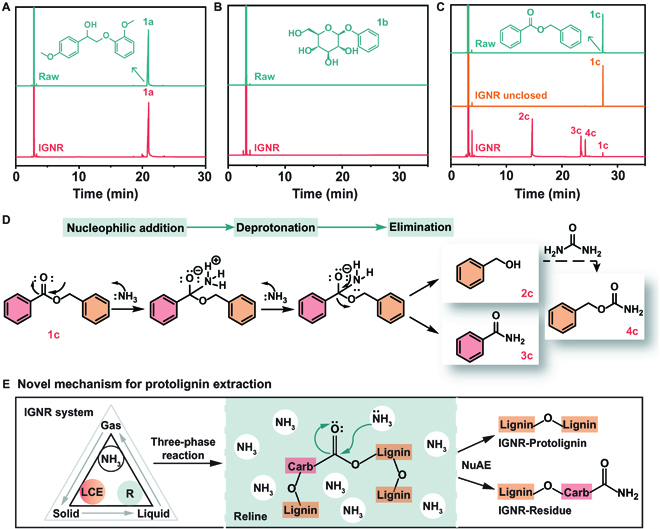
Mechanism verification by model compound studies. (A to C) GC results of (A) 2-(2-methoxyphenoxy)-1-(4-methoxyphenyl) ethanol (1a), (B) phenyl β-d-glucopyranoside (1b), and (C) benzyl benzoate (1c) before and after reaction in the IGNR system; the products were identified by GC-MS after extraction workup. (D) Nucleophilic addition–elimination mechanism for 1c cleavage. (E) Protolignin extraction pathway in the 3-phasic IGNR system. LCE, lignin–carbohydrate ester; R, reline; NuAE, selective nucleophilic addition–elimination; Carb, carbohydrates.

It was observed from the gas chromatography (GC) results that only 1a was detected after the reaction, demonstrating the good stability of lignin β-O-4′ linkages in the IGNR system (Fig. 3A). When 1b was chosen as the model compound of lignin–carbohydrate ether, no monomeric products (e.g., cleaved phenol) were detected after the reaction under the same conditions (Fig. 3B). This indicates that the lignin–carbohydrate ether was also stable in the IGNR system. When 1c was used as the model compound of lignin–carbohydrate ester in the IGNR system, it was seen that the reactant 1c was obviously reduce while benzyl alcohol (2c), benzamide (3c), and benzyl carbamate (4c) were detected by GC and GC-MS (mass spectrometry) (Fig. 3C and Fig. S1). It is worth noting that when 1c was reacted in an open flask (without pressurized NH_3_), almost no conversion of 1c was observed, revealing the specific impact of NH_3_ on the lignin–carbohydrate ester in the IGNR system. Based on the product distributions from the model compound studies, we propose that the mechanism for selective cleavage of lignin–carbohydrate ester was mainly achieved through nucleophilic addition–elimination (Fig. 3D). Urea in the IGNR system was partially decomposed into NH_3_ at 150 °C, while NH_3_ acted as a nucleophile to attack the carbonyl carbon in the lignin–carbohydrate ester, cleaving the π bond and forming a tetrahedral intermediate. To restore the double bond, the benzyloxy group was eliminated to form benzamide (3c). At the same time, the removed benzyloxy group was alkaline enough to obtain protons, forming the major product of 2c, while the minor product 4c was likely formed from the reaction of 2c and urea.

In summary, these verifications by model compounds demonstrated the mechanism for protolignin extraction via the selective cleavage of lignin–carbohydrate ester (Fig. 3E). IGNR was a 3-phasic closed-loop system, in which reline as a liquid phase acted as both the solvent and the nucleophile generator. The in situ generated NH_3_ gas attacked the lignin–carbohydrate ester in lignocellulose, and the liberated lignin fragments were solubilized in reline for extraction. This allowed the system to precisely cleave the lignin–carbohydrate esters in the LCCs while simultaneously establishing a protective system for the ether bond in the lignin, achieving one-pot protolignin extraction from lignocellulose.

### One-pot protolignin extraction from lignocellulose

**Fig. 4.**
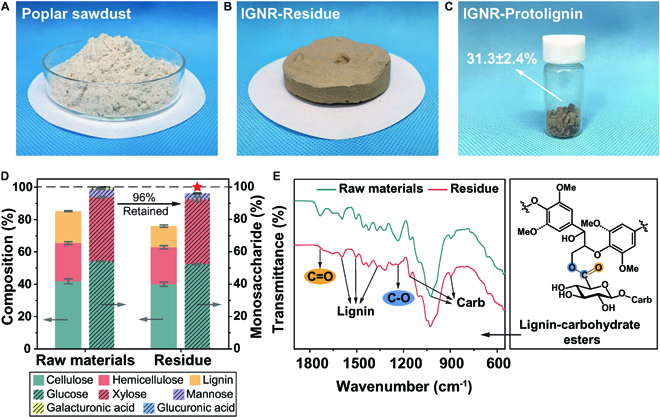
One-pot protolignin extraction and characterizations of the residue. (A to C) Photographs of (A) poplar sawdust, (B) IGNR–Residue, and (C) IGNR–Protolignin. (D) Compositional analysis results. (E) FTIR analysis of the raw material and IGNR–Residue.

Based on the studies using model compounds, one-pot protolignin extraction from poplar wood sawdust was carried out in the IGNR system at 150 °C for 6 h with a 1:10 mass ratio of poplar sawdust (Fig. 4A) to solvent (10 and 100 g, respectively). After IGNR fractionation and lignin extraction, the average yield of IGNR–Protolignin was 31.3 ± 2.4 wt%, which was comparable to the theoretical lignin–carbohydrate ester content in poplar (~30%), indicating that the proposed reaction can liberate lignin–carbohydrate ester-linked lignin. It can be seen that whitish poplar sawdust became a light brown residue after fractionation (Fig. 4B), and the obtained IGNR–Protolignin was also light brown (Fig. 4C).

Compositional and functional group analyses of IGNR–Residue were carried out to verify the effect of IGNR fractionation on the carbohydrates [46]. From the composition analysis, lignin content decreased from 19.8 wt% to 13.2 wt% (~33% lignin removal) after IGNR fractionation, while cellulose and hemicellulose contents in IGNR–Residue showed high retentions of 95.5% and 97.1%, respectively (Fig. 4D and Table S1). The total retention of monosaccharides in IGNR–Residue was 96% (Fig. 4D and Table S2). The glucose content remained almost unchanged at 96.7%, while an even higher proportional content of xylose than the raw material was observed. Compared to the control, 68.6% of mannose was retained, while less was found for galacturonic acid and glucuronic acid. This is likely because some of the hemicellulose side chains exposed after the reaction underwent degradation or neutralization in the alkaline aqueous solution [47].

Meanwhile, Fourier transform infrared (FTIR) analysis was performed on the raw material and IGNR–Residue. The spectrum of the raw material (Fig. 4E) shows the typical absorption peaks of various functional groups in cellulose, hemicellulose, and lignin [44]. Notably, the stretching vibration peaks of nonconjugated C=O ester bond at 1,735 cm^−1^ and the C–O ester bond at 1,234 cm^−1^ almost disappeared after the reaction, proving that the lignin–carbohydrate ester was effectively cleaved by the IGNR system [48–50]. In summary, the one-pot protolignin extraction from lignocellulose was realized according to the nucleophilic addition–elimination mechanism, as demonstrated in the previous study of model compounds. The dissociation of lignin–carbohydrate esters and extraction of protolignin were precise and targeted, and the reaction process had minimal effect on the carbohydrates.

### Structural elucidation of IGNR–Protolignin

**Fig. 5.**
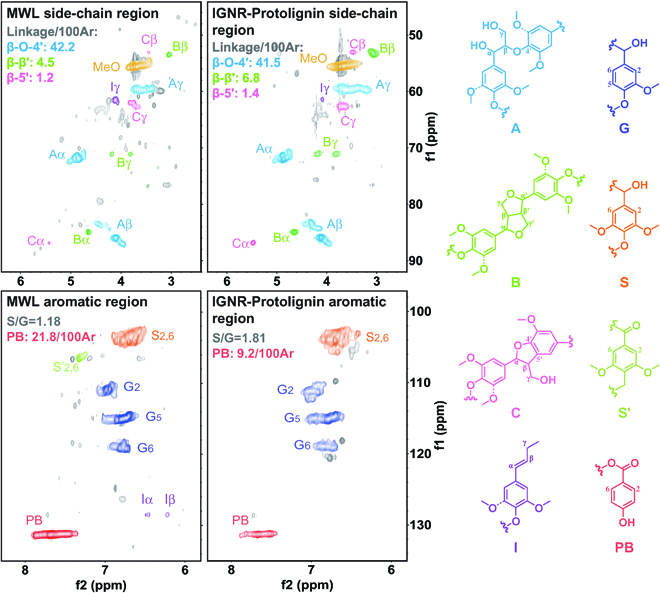
Elucidation of the interlinkages of MWL and IGNR–Protolignin by 2D HSQC-NMR spectroscopy.

To investigate the interlinkages of IGNR–Protolignin, MWL was used as the typical protolignin for comparison. Both the samples were characterized by 2-dimensional heteronuclear single quantum coherence–nuclear magnetic resonance (2D HSQC-NMR) spectroscopy. The side-chain and aromatic regions of MWL and IGNR–Protolignin are shown in Fig. 5. The typical interlinkages and cross signals in the IGNR–Protolignin spectrum were almost the same as those in MWL. In the side-chain region, IGNR–Protolignin behaved similarly to MWL in the highest proportional β-O-4′ structure (A, β-O-4′, C_α_-H_α_ at δ_C_/δ_H_ = 71.5/4.87, C_β_-H_β_ at δ_C_/δ_H_ = 85.9/4.12 and 83.9/4.29, C_γ_-H_γ_ at δ_C_/δ_H_ = 59.5 to 59.7/3.40 to 3.63) and in less abundant resinol substructures (B, β-β′, C_α_-H_α_ at δ_C_/δ_H_ = 84.9/4.67, C_β_-H_β_ at δ_C_/δ_H_ = 53.5/3.06, C_γ_-H_γ_ at δ_C_/δ_H_ = 71.0/3.83 and 4.18) and phenylcoumaran substructures (C, β-5′, C_α_-H_α_ at δ_C_/δ_H_ = 86.8/5.46, C_β_-H_β_ at δ_C_/δ_H_ = 53.3/3.46, C_γ_-H_γ_ at δ_C_/δ_H_ = 62.5/3.73). Also, both IGNR–Protolignin and MWL showed structural signals associated with the p-hydroxycinnamyl alcohol end groups (I, C_γ_-H_γ_ at δ_C_/δ_H_ = 61.4/4.10). In the aromatic region, the signals of both syringyl (S, C_2,6_-H_2,6_ at δ_C_/δ_H_ =103.8/6.71) and guaiacyl units (G, C_2,5,6_-H_2,5,6_ at δ_C_/δ_H_ = 111.2/6.98, 114.9/6.81, and 119.0/6.80) were pronounced and almost free of polycondensation. Unlike MWL, the *p*-hydroxybenzoate substructures (PB; C_2,6_-H_2,6_ at δ_C_/δ_H_ = 131.3/7.62) showed a slightly reduced content (from 21.8/100Ar to 9.2/100Ar) in IGNR–Protolignin. This can be explained by the effective disruption of the ester bond in the PB unit during nucleophilic addition–elimination, resulting in a weakened PB signal. It should be noted that *p*-hydroxybenzamide was also identified after extraction of the fractionated mixture (Fig. S2). These results were strong evidence that the IGNR system can perform the precise dissociation of lignin–carbohydrate esters.

The number of linkages between the basic units was semiquantitatively calculated by HSQC. The results showed that the number of various linkage bonds in IGNR–Protolignin was close to that in MWL: the content of dominant β-O-4′ in MWL and IGNR–Protolignin was 42.2 and 41.5 per 100 aromatic units [51], respectively. The respective contents of β-β′ and β-5′ linkages in MWL (4.5/100Ar and 1.2/100Ar) and IGNR–Protolignin (6.8/100Ar and 1.4/100Ar) were also nearly the same. IGNR–Protolignin was found to be highly similar to MWL in terms of linkage types and content, revealing their similarities in natural structure. In the aromatic region, the S/G value of IGNR–Protolignin (1.81) was higher than that of MWL (1.18). The thioacidolysis of lignin was further carried out to determine the S/G ratio (Fig. S3), with values of 4.9 and 2.3 observed for IGNR–Protolignin and MWL, respectively. This is due to the fact that the γ-ester bond was more attached to the C_γ_ position of the syringyl units, leading to easier isolation of high S-ratio lignin in case of the dissociation of ester bonds [52,53]. When further catalytic depolymerization was performed on IGNR–Protolignin and MWL according to the literature [54], the results revealed that higher yield of propylphenol monomers was achieved compared with that from MWL (Fig. S4). The pyrolyzed monomeric products and functional groups of IGNR–Protolignin were also nearly identical to those of MWL (Fig. S6 and Tables S3 and S4), which demonstrated the superior activity of IGNR–Protolignin for downstream lignin depolymerization. Further characterization of FTIR showed that the functional groups and core structures of IGNR–Protolignin and MWL were similar (Fig. S6). It was noteworthy that when MWL was treated in the IGNR system under the same condition, there was little difference of MWL before and after treatment in terms of β-O-4′ content and structure (Figs. S7 and S8). This observation was consistent with the model compound studies in Fig. 3A.

### Molecular weight determination

**Fig. 6.**
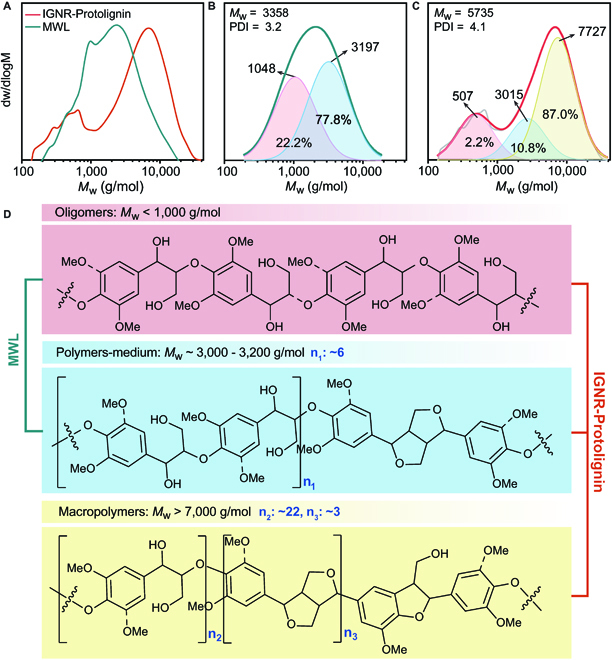
Molecular weight determination of lignin fractions. (A) Molecular weight determination of MWL and IGNR–Protolignin by GPC. (B and C) Fitted GPC curves of (B) MWL and (C) IGNR–Protolignin. (D) Proposed substructures of different lignin fragments. Note: n_1_, n_2_, and n_3_ represent the repeating unit in medium polymers and β-O-4′ and β-β′ in macro polymers, respectively in the proposed lignin substructures. The values are estimated according to the molecular distribution and semiquantitative analysis by 2D HSQC NMR.

The molecular weights of MWL and IGNR–Protolignin were tested separately by gel permeation chromatography (GPC; Fig. 6A and Table S5), and the weight-average molecular weight (*M*_w_) of IGNR–Protolignin (5,735 g/mol) was higher than that of MWL (3,358 g/mol). The polydispersity index of IGNR–Protolignin was also larger than that of MWL (4.1 versus 3.2). The GPC curves of the 2 lignin species were fitted using the log-normal distribution function for peak differentiation imitation, and the results are shown in Fig. 6B and C. The peak *M*_w_ values of the 2 main peaks of MWL were 1,048 g/mol (pink area) and 3,197 g/mol (blue area), and the ratio of oligomeric and medium lignin fractions was calculated as 22:78. The peak *M*_w_ values of the 3 main peaks fitted in IGNR–Protolignin were 507 g/mol (pink area), 3,015 g/mol (blue area), and 7,727 g/mol (yellow area). High-molecular-weight lignin fractions were predominated (~87%) in IGNR–Protolignin. Possible substructures of IGNR–Protolignin are proposed according to the in-depth GPC and HSQC NMR analysis as shown in Fig. 6D. Unlike conventionally extracted protolignin, the IGNR system enabled the IGNR-Protolignin across a broad range of molecular weights.

### Condition optimizations of IGNR–Protolignin

**Fig. 7.**
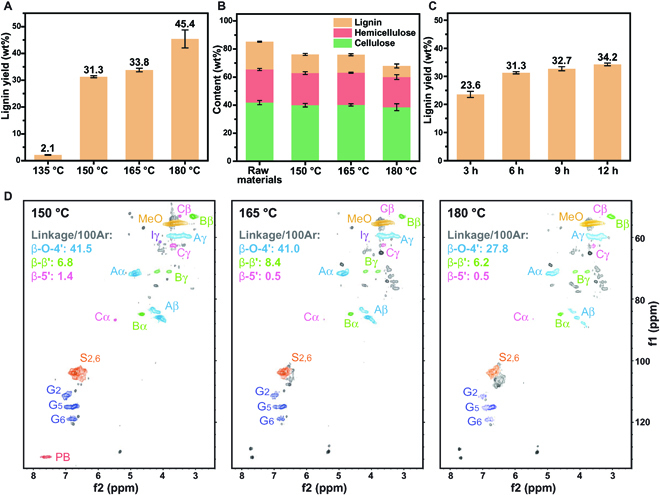
Optimization of reaction conditions for IGNR–Protolignin extraction. (A) Lignin yield and (B) compositional content of the residual solids under different extraction temperatures. (C) Obtained yield of lignin under different reaction time at 150 °C. (D) Structural characterization of the extracted lignin by 2D HSQC NMR.

For the reaction condition considerations, we found that the in situ generated NH_3_ from urea decomposition in reline was crucial for the protolignin extraction, and the effect of temperature and time was non-ignorable. Therefore, a detailed optimization of the reaction conditions as shown in Fig. 7, was performed and analyzed, i.e., temperatures of 135, 150, 165, and 180 °C and reaction times of 3, 6, 9, and 12 h. It was found that 135 °C led to little amount of lignin extraction as the temperature was not sufficient for NH_3_ generation from urea. At 150 °C, the lignin yield increased to around 31.3 wt% and the obtained lignin demonstrated native structures as protolignin. In the meantime, the total retention of cellulose and hemicellulose in the residual solid was as high as 96.1% and the lignin removal was 33.3%. The excellent mass balance of lignin and carbohydrates indicated that the protolignin was extracted by targeting cleavage of lignin–carbohydrate esters. The yield of lignin increased a bit when the temperature increased to 165 °C, and the pressure of the reaction also increased, which indicated that more NH_3_ was generated. It was found from the structural analysis that the obtained lignin was still with native lignin substructures, albeit the content of β-O-4′ (41.0/100Ar) decreased a bit and extra minor condensation of signal S-type units was observed as shown in Fig. 7D. The PB substructures disappeared possibly due to the intensification of the nucleophilic addition–elimination reaction with more NH_3_ at higher temperature. A further increased temperature of 180 °C led to a reduced content of β-O-4′ (27.8/100Ar) and more severe condensation in the extracted lignin, albeit the lignin yield increased to 45.4 wt%. Nevertheless, reaction temperatures of 165 and 180 °C also demonstrated high (>92%) carbohydrate retention, indicating that the IGNR system had little effect on the cellulose and hemicellulose components even at higher temperatures. Possibly other linkages such as ether bonds in lignocellulose were cleaved at higher temperature. Therefore, with the focus of protolignin extraction with native substructures, a temperature of 150 °C is relatively appropriate for the IGNR system.

The optimizations of reaction time were carried out at 150 °C for 3, 6, 9, and 12 h, respectively. The yields of IGNR–Protolignin were correspondingly 23.6, 31.3, 32.7, and 34.2 wt%. It was found from the results that yields of IGNR–Protolignin were comparable except that lower yield was obtained for 3 h. Therefore, 6 h was chosen as the optimal reaction time from the view of moderate yield and lower energy consumption. In summary, at 150 °C, 6 h was chosen as the optimal conditions for the IGNR–Protolignin extraction in this work. Nevertheless, it was suggested that a temperature range of 150 to 165 °C was also applicable for IGNR–Protolignin extractions.

### Up-scaled extraction of IGNR–Protolignin

**Fig. 8.**
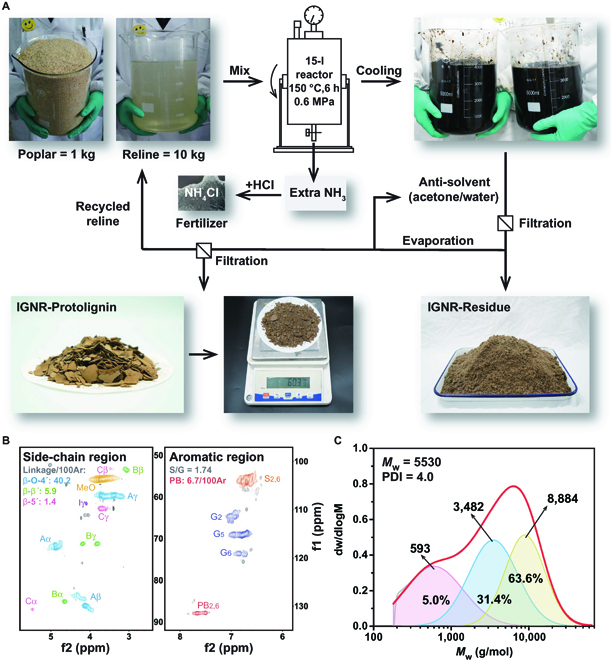
Procedures and characterizations for up-scaled extraction of IGNR–Protolignin. (A) Flow diagram of the 100-fold up-scaled poplar sawdust fractionation for IGNR–Protolignin extraction. (B) 2D HSQC-NMR analysis of IGNR–Protolignin in large quantities. (C) Molecular weight and distribution of IGNR–Protolignin by GPC.

To demonstrate the feasibility of the IGNR system for larger-scale protolignin extraction, 1 kg of poplar sawdust (100-fold increase) and 10 kg of reline were mixed in a 15-l sealed industrial pulping reactor (Fig. 8A). The reactor was heated to 150 °C to form the IGNR system, and the in situ generated NH_3_ pressure was ~0.6 MPa. After reaction for 6 h, the mixture was cooled naturally. The separated lignin was dissolved by acetone antisolvent solution while sealed, and then the acetone was removed from the lignin solution by rotary evaporation. IGNR–Protolignin was finally precipitated by adding an appropriate amount of water. Excess generated NH_3_ could be converted to NH_4_Cl as fertilizer (Fig. S9).

A total of ~60 g of IGNR–Protolignin was obtained after drying, which is consistent with its lignin yield for a small batch reaction. 2D HSQC-NMR structural characterization on the obtained IGNR–Protolignin was performed (Fig. 8B), and the characteristic signals were very similar to those of the previous small-batch IGNR–Protolignin. The β-O-4′, β-β′, and β-5′ contents in the side-chain region were 40.2/100Ar, 5.9/100Ar, and 1.4/100Ar, respectively. The S/G ratio was calculated to be 1.74, and *M*_w_ was 5,530 g/mol (Fig. 8C), which were also consistent with the data in the small-batch IGNR–Protolignin extraction. These results suggest that the nucleophilic addition–elimination mechanism of IGNR is robust and IGNR–Protolignin can be produced in large quantities or even at an industrial scale.

### Solvent recyclability and universality

To evaluate the recyclability of the IGNR system, the reline solvent was recycled and reused (Figs. S10 to S12). First, the ^13^C NMR spectrum results (Fig. S10) showed that no obvious compositional and structural changes in reline recovered after one reaction compared to the fresh reline, except for a slight decrease in urea content. Furthermore, the results of lignin extraction showed that reline could be reused at least 3 times, and the yields of IGNR–Protolignin and IGNR–Residue were similar to those of the pristine IGNR system (Fig. S11). Structural analysis of the recycled IGNR–Protolignin showed a slight decrease in β-O-4′ content, which remained generally high (above 30/100Ar) as the number of recoveries increased (Fig. S12). This was possibly due to the amount of urea in reline decreasing after several cycles and limitations in the nucleophilic addition–elimination. Therefore, reline was recovered for the third time and extra 50% urea was replenished to guarantee the sufficient nucleophilic generator for IGNR fractionation. As a result, the β-O-4′ content increased and reached 34.2/100Ar. In summary, the IGNR system can be recovered and recycled several times without losing its effectiveness, suggesting promising robustness for possible industrial application.

For the applicability of the IGNR system on different lignocellulosic resources, additional 4 types of lignocellulose from birch, pine, bamboo, and wheat straw were used to perform protolignin extraction under the same conditions as poplar IGNR–Protolignin extraction. It was found that all IGNR–Residues displayed similar appearances. Figure S13 showed the interlinkages of birch, pine, bamboo, and wheat straw IGNR–Protolignins by 2D HSQC NMR. The yield of IGNR–Protolignin–birch was about 21.5 wt%, and it retained high β-O-4′ content of 51.8/100 Ar. Meanwhile, IGNR–Protolignin–birch was similar to that of poplar, with an intact lignin structure and the number of β-β′ and β-5′ reaching 6.5/100Ar and 1.48/100Ar, respectively. The S-type signal in the aromatic region was also almost free of condensation. A lower yield (8.0 wt%) of IGNR–Protolignin–pine was obtained with content of 19.6/100Ar for β-O-4′ linkage, 1.26/100Ar for β-β′ linkage, and 7.59/100Ar for β-5′ linkage. The lower yield and β-O-4′ content of IGNR–Protolignin–pine were possible because pine lignocellulose has more complex lignin structure and limited amount of lignin–carbohydrate esters [36]. Similarly, IGNR–Protolignin–bamboo exhibited high β-O-4′ content and no condensation in the aromatic region. Meanwhile, both the original ferulate and *p*-coumarate (*p*CA) substructures in the bamboo lignocellulose disappeared, further revealing the effectiveness of the IGNR system. However, it was found that the raw bamboo lignocellulose contained non-acid-degradable silica ash that could hardly be removed, resulting in a low lignin yield of 12.9 wt% after calculation. In addition, the yield of IGNR–Protolignin–wheat straw was 20.8 wt% and the lignin was not condensed, with only a small amount of *p*CA substructure left. On the whole, the preliminary results demonstrated promising applicability of the IGNR system on various biomasses. Additionally, it should be noted that the lignin–carbohydrate ester content varied among different lignocelluloses. The effectiveness of the IGNR system when using other lignocelluloses still requires further study, especially for lignin–carbohydrate ester-rich or genetically modified plant-derived lignocelluloses.

## Discussion

This work mainly focused on the protolignin extraction by targeting lignin–carbohydrate ester cleavage. An IGNR system was designed, and the whole system showed perfect cooperation. Although reline was simply a liquid solvent at room temperature, the urea in the solvent started to decompose to NH_3_ when the temperature increased to 150 °C. NH_3_ was the most important link in the system, which performed the most important task of breaking the ester bond as a nucleophilic reagent. Solid lignocellulose was dispersed in reline, and the liquid reline provides a strong hydrogen bonding system necessary to help deconstruct lignocellulose and dissolve the lignin. We specifically compared IGNR–Protolignin with MWL in terms of reaction time, lignin yield, molecular weight, β-O-4′, and monomer yield after depolymerization, and the results are shown in Table S6 and Fig. S14. It can be concluded that IGNR–Protolignin is similar to MWL in terms of the β-O-4′ content and depolymerized monomer yield. But the lignin yield and molecular weight of IGNR–Protolignin are obviously higher than those of MWL. Meanwhile, the reaction time is shorter and the steps are more concise. Therefore, the IGNR method can achieve higher separation efficiency and lower energy consumption than the MWL method, which is conducive to improving yield and reducing cost, and is expected to achieve the target of large-scale production eventually. By comparing the protolignins extracted by different methods (Table S7), we proved that IGNR–Protolignin has not only a similar β-O-4′ content to MWL and CEL but also unprecedented advantages in terms of extraction efficiency. Notably, IGNR–Protolignin enriched the protolignin family and induced a better understanding of lignin structure elucidation and valorization.

Unlike previous strategies for protolignin extraction, the effect of the extraction process on the carbohydrate components was minimal, and only ester-linked lignin was extracted. Therefore, the rest of the ether-linked lignin as well as carbohydrates remained in the solid residues. Therefore, the IGNR system could be easily integrated with current biorefinery strategies to achieve full component utilization. For example, the diol-stabilized system report can be used to isolate the rest of ether-linked lignin and subsequently valorize the carbohydrate components [27,30,55]. In addition, the IGNR residue can also be subjected to an aldehyde-stabilized strategy to isolate lignin before enzymatic digestion [25,26], or it can be directly subjected to a reduction-catalyzed depolymerization operation [54,56]. Additionally, the content of *p*-hydroxybenzoate interlinkages decreased after IGNR treatment. By a simple extraction of the recycled reline, *p*-hydroxybenzamide was identified using GC-MS (Fig. S2). Therefore, the IGNR system can provide a new route for directly accessing amide from biomass or synthesis of amides from esters.

## Materials and Methods

### Methods

***Preparation of reline.*** Reline was synthesized from the mixture of choline chloride and urea (molar ratio 1:2) at 80 °C with stirring until a homogeneous clear liquid appeared.

***Model compound verification in the IGNR system.*** Three model compounds, 2-(2-methoxyphenoxy)-1-(4-methoxyphenyl) ethanol (1a), phenyl β-d-glucopyranoside (1b), and benzyl benzoate (1c) (0.1 g), were added to reline (20 g) and reacted at 150 °C for 6 h in a closed autoclave (YZ-100, Shanghai Yanzheng Experimental Instrument Co. Ltd., Shanghai). Another reaction in an unclosed round-bottom flask was used as the control. Next, 10 g of the cooled reaction product was extracted by adding ethyl acetate (15 ml, 3 times) and water (15 ml), and the ethyl acetate phase was concentrated under reduced pressure and dried. The product was diluted in ethyl acetate (2 ml) through a 0.22-μm polytetrafluoroethylene filter and analyzed by GC-MS and GC-FID (Flame Ionization Detector).

***IGNR–Protolignin extraction.*** First, 10 g of poplar sawdust was added to 100 g of reline in a sealed autoclave and heated at 150 °C for 6 h with continuous stirring. After cooling to room temperature, the mixture was dissolved in a 10-fold volume of acetone and water mixture (7:3 by volume) with stirring, and the insoluble residues were filtered out and dried. Then, the mixed solution was rotary evaporated under reduced pressure to remove recyclable acetone. IGNR–Protolignin was precipitated from the remaining aqueous solution and dried at 45 °C for further characterization. MWL was extracted from lignocellulose for comparison, and detailed procedures were performed according to the literature [17,27].

***Scaled-up extraction of IGNR–Protolignin.*** The extraction of IGNR–Protolignin up-scaled 100-fold was performed with the same operating steps as for the initial reaction. Scaled experiments were conducted using 1 kg of poplar sawdust and 10 kg of reline in a 15-l sealed cooking reactor equipped with a pressure detector (P001, China National Pulp and Paper Research Institute Co. Ltd., Beijing). The reactor was programmatically heated to 150 °C and reacted for 6 h. Pressurized NH_3_ inside the reactor was released before opening the reactor. Excess unreacted NH_3_ was released and stabilized in aqueous hydrochloric acid, affording ammonium chloride after concentration and crystallization.

### Characterizations

***Composition analyses of the insoluble residue.*** The compositions of the insoluble residue were measured according to the National Renewable Energy Laboratory procedure. The monosaccharides were detected by high-performance liquid chromatography (HPLC; Agilent 1260 Infinity II, USA). An Aminex column HPX-87H (300 × 7.8 mm, Bio-Rad) was used to analyze the monosaccharides at 50 °C with 5 mM sulfuric acid at 0.6 ml/min. Under these conditions, xylose, mannose, and galactose were eluted at the same retention time, and the detailed monosaccharide moieties in the residue were tested by high-performance anion exchange chromatography (HPAEC; Dionex ICS3000, USA). The sample was treated with 6% H_2_SO_4_ at 105 °C for 2.5 h and then filtered, diluted 100-fold before the HPAEC analysis. Calibration was performed with standard solutions of d-glucose, d-xylose, d-mannose, glucuronic acid, and galacturonic acid.

***2D HSQC-NMR spectroscopy.*** First, 60 mg of lignin was dissolved in 0.6 ml of dimethyl sulfoxide (DMSO)-d_6_. All spectra were acquired on a Bruker AVIII HD 500 spectrometer (Bruker Co., Karlsruhe, Germany). The spectral widths were 5,000 and 20,000 Hz for the ^1^H and ^13^C dimensions. A total of 1,024 collected points were used for the ^1^H dimension with a recycle delay of 1.5 s, while 64 and 256 transients were used for the ^13^C dimension. The DMSO-d_6_ peak was applied as internal chemical shift reference point (δ_C_/δ_H_ = 39.52/2.50).

***GPC analysis.*** Dried acetylated lignin samples were dissolved in tetrahydrofuran (2 mg/ml), filtered through 0.22-μm polytetrafluoroethylene filters, and analyzed by HPLC. The columns used were PLgel 5 μm Guard (50 × 7.5 mm), PLgel 5 μm MIXED-C (300 × 7.5 mm), and PLgel 3 μm MIXED-E (300 × 7.5 mm) in tandem, and the injection volume was 20 μl. The *M*_w_ value of lignin was calculated using the polystyrene standard sample with average *M*_w_ of 640, 1,230, 1,880, 4,720, 6,580, 9,580, 22,130, and 27,500 (g/mol) as standard curves. *M*_w_ was calculated using Agilent GPC software, the GPC curve was split by Peakfit software, and the log-normal amp was used for fitting to obtain the experimental results.

## Data Availability

The data that support the findings of this study are available from the corresponding author upon reasonable request.

## Supplementary Materials

Supplementary Materials Experimental Procedures Figs. S1 to S14 Tables S1 to S7 References (57–67)
