# Supplementary material for: One-Pot Protolignin Extraction by Targeted Unlocking Lignin–Carbohydrate Esters via Nucleophilic Addition–Elimination Strategy
Source: Research (Wash D C). 2023 Mar 9;6:0069. doi: 10.34133/research.0069 (PMC10013968; doi:10.34133/research.0069)
Supplement: Supplementary Materials — Experimental Procedures Figs. S1 to S14 Tables S1 to S7 References (57–67) [file research.0069.f1.docx]

**Supplemental Information**

**One-pot Protolignin Extraction by Targeted Unlocking Lignin–Carbohydrate Esters via Nucleophilic Addition-Elimination Strategy**

Yuhan Lou, Xinyue Sun, Yanyan Yu, Suqing Zeng, Yilin Li, Yongzhuang Liu,^*^ Haipeng Yu^*^

Key Laboratory of Bio-Based Material Science and Technology of Ministry of Education, Northeast Forestry University, Harbin 150040, P. R. China.

*Correspondence: [yuhaipeng20000@nefu.edu.cn](mailto:yuhaipeng20000@nefu.edu.cn), [lyz@nefu.edu.cn](mailto:lyz@nefu.edu.cn)

| **Table of Content** | **Page** |
| --- | --- |
| Supplemental Experimental Procedures | S1 |
| Supplemental Figure S1-S14 | S2-S15 |
| Supplemental Table S1-S7 | S16-S22 |
| Supplemental References | S23 |

Supplemental experimental procedures

- 1. **Materials**

All chemicals were purchased from Aladdin, TCI, Rhawn, and Fuyu chemicals without further purification. Choline chloride and urea (analytical reagent) were used for reline preparation. Model compounds 1a-1c were used for reaction mechanism analysis. Specifically, 2-(2-methoxyphenoxy)-1-(4-methoxyphenyl) ethanol (1a) was synthesized according to the literature^1^. Pyridine, acetic anhydride, methanol, ethanol, and tetrahydrofuran (chromatography purity) were applied to lignin acetylation and molecular weight analysis. Poplar (*Populus ussuriensis*) sawdust was provided by a local company in Harbin. The 60-mesh wood powder was used as a small batch experiment, and no particle screening was performed for the up-scaled extraction experiment. The MWL was extracted by following from published steps^2,3^. The lignin samples for thioacidolysis were prepared according to the literature^4,5^.

**1.2 Semi-quantitative analysis of substructures by 2D HSQC NMR**

2D HSQC NMR was used for semi-quantitative analysis of the obtained lignins, relative content of the substructures was described as content per 100 aromatic units^6^. Detailed calculations after integral were as follows:

$Total aromatic rings=\left( \frac{S_{2/6}+{S'}_{2/6}}{2} \right)+\left( \frac{G_{2}+G_{5}+G_{6}}{3} \right)+\left( \frac{\mathrm{PB}_{2/6}}{2} \right)$

$$Ratio S (per 100Ar)=\left( \frac{S_{2/6}+{S'}_{2/6}}{2\times Total aromatic rings} \right) \times100$$

$$Ratio G (per 100Ar)=\left( \frac{G_{2}+G_{5}+G_{6}}{3\times Total aromatic rings} \right) \times100$$

$$Ratio PB (per 100Ar)=\left( \frac{\mathrm{PB}_{2/6}}{2\times Total aromatic rings} \right) \times100$$

$$S/G=\frac{Ratio S}{Ratio G}$$

$$\beta-O-4 linkages=\left( \frac{{\beta-O-4'}_{\alpha}}{Total aromatic rings\times1.3} \right) \times100$$

$$\beta-\beta linkages=\left( \frac{{\beta-O-4'}_{\alpha}}{Total aromatic rings\times1.3} \right) \times100$$

$$\beta-5 linkages=\left( \frac{{\beta-O-4'}_{\alpha}}{Total aromatic rings\times1.3} \right) \times100$$

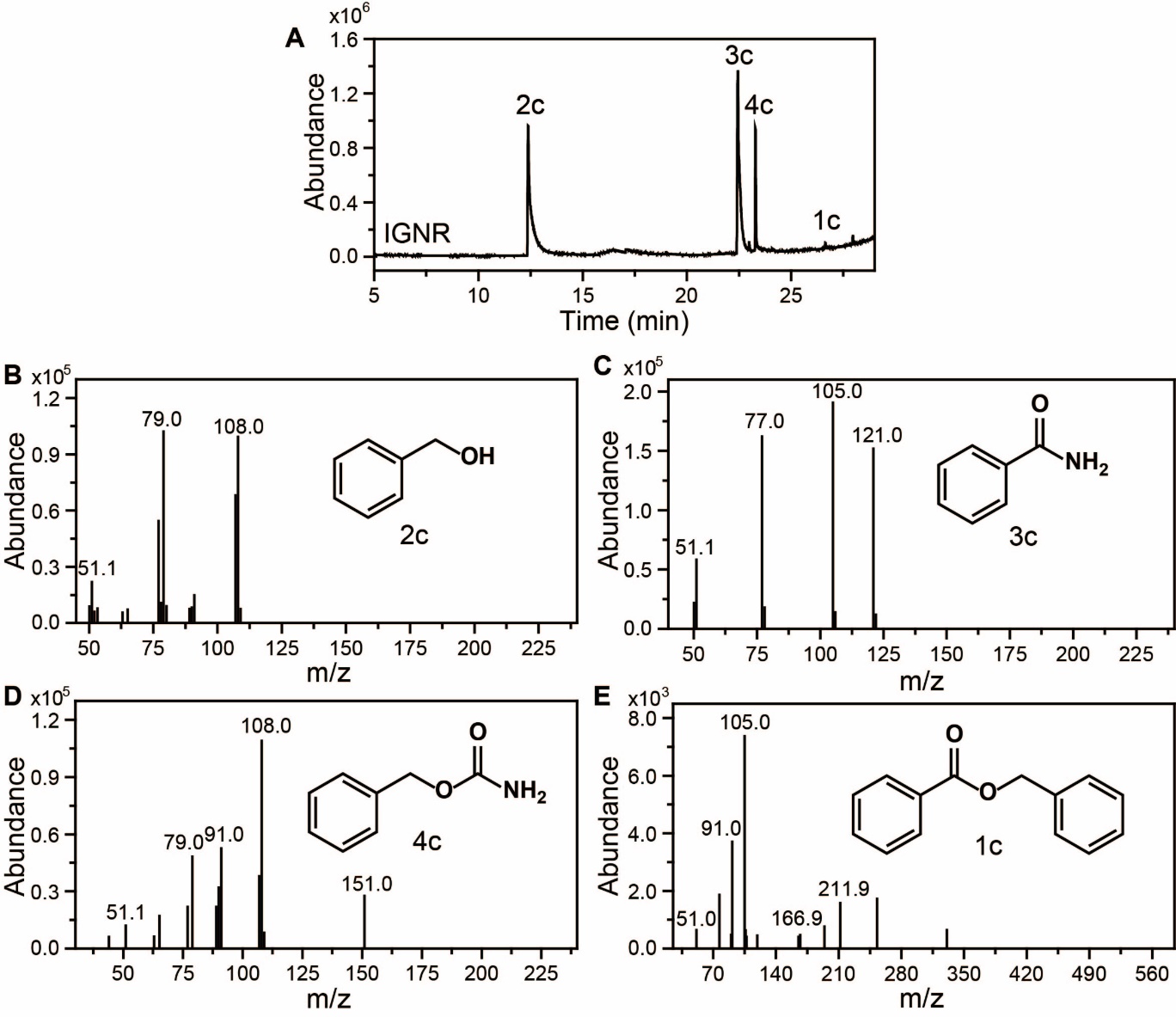


**Figure S1. GC-MS spectra of confirmed products from decomposing model compound 1c in IGNR system**


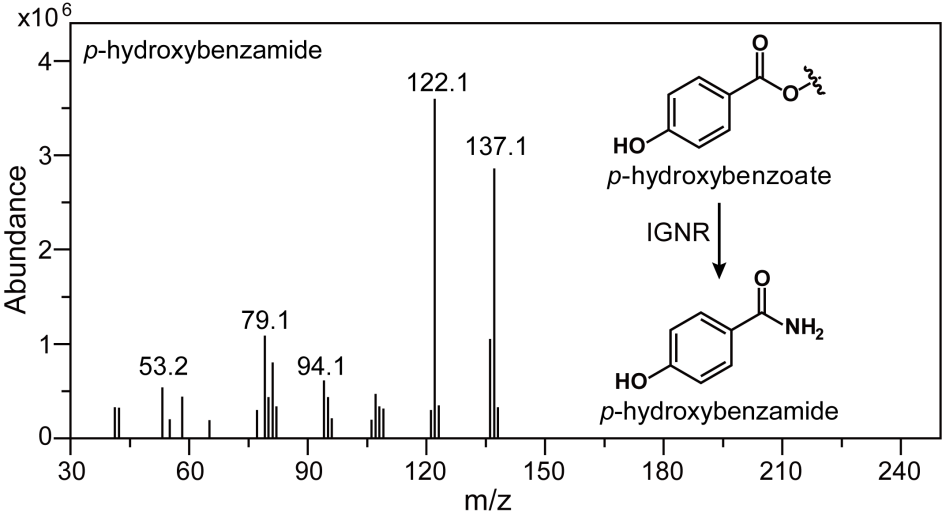


**Figure S2. Mass spectrum of confirmed *p*-hydroxybenzamide in the recycled reline**

**
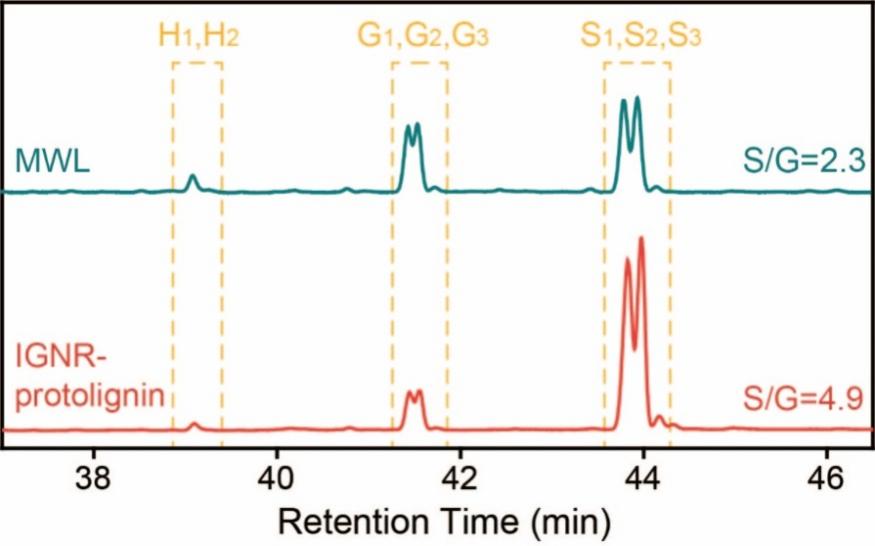
**

**Figure S3. GC spectra of thioacidolysis products from MWL and IGNR-Protolignin**


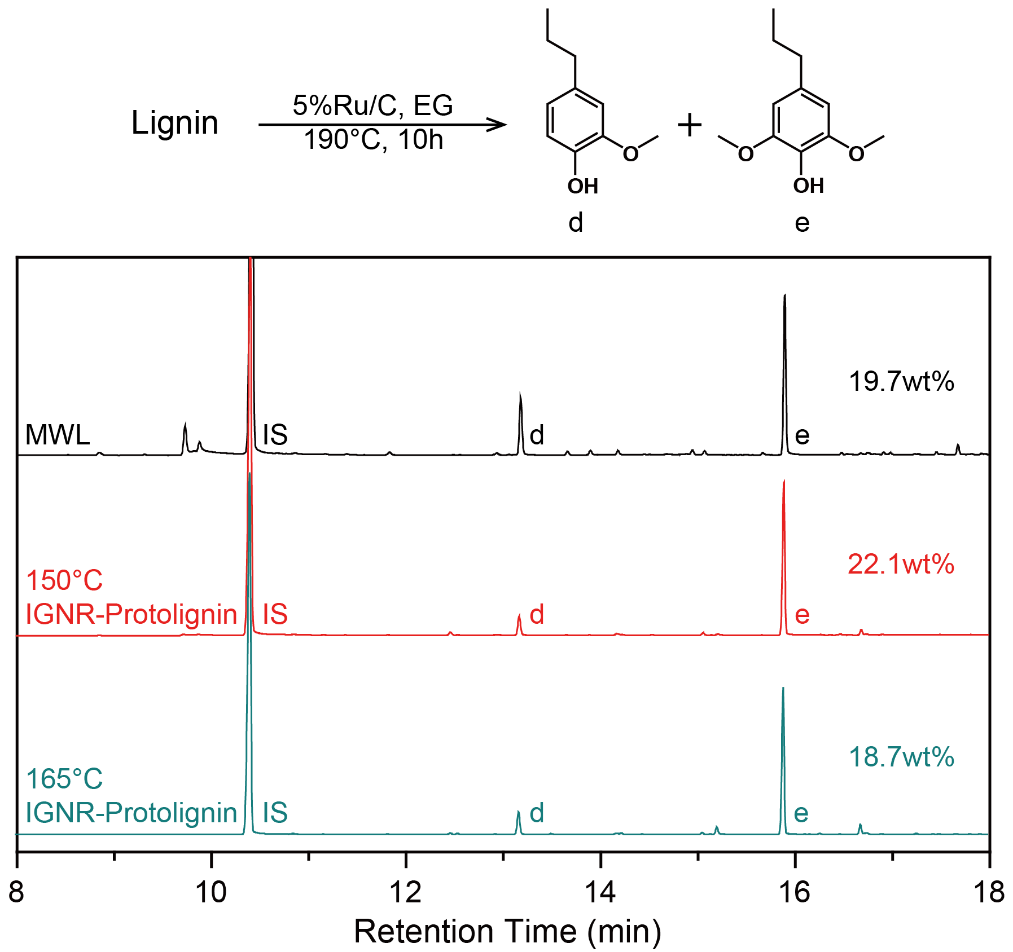


**Figure S4. Reductive catalytic fractionation of MWL and IGNR-Protolignin**

We carried out reductive catalytic depolymerization (RCF) of MWL and IGNR-Protolignin according to a recent RCF approach in literature.^7^ The lignin samples were respectively catalyzed using 5% Ru/C in the ethylene glycol solvent and reacted at 190 °C for 10 h. The total yields of 4-propylguaiacol and 4-propylsyringol were calculated. It was found that the monomer yield of IGNR-Protolignin was 22.1 wt% which was higher than the yield (19.7 wt%) of monomers from MWL. These results demonstrated that IGNR-Protolignin had higher reactivity and was promising for further downstream valorization. With further optimization of the protolignin extraction conditions, extra depolymerization of IGNR-Protolignin at 165 °C was also performed, and the monomer yield was 18.7 wt% which also revealed the good reactivity.


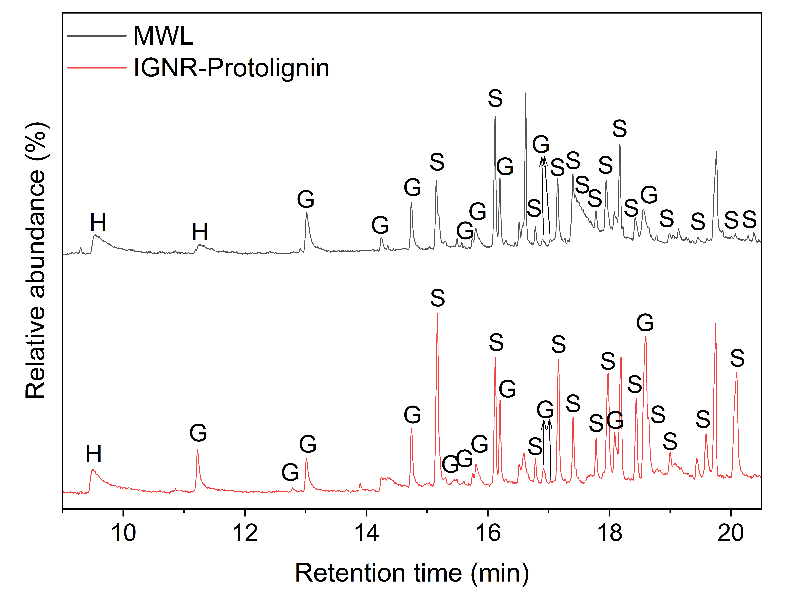


**Figure S5. Py-GC-MS of MWL and IGNR-Protolignin**


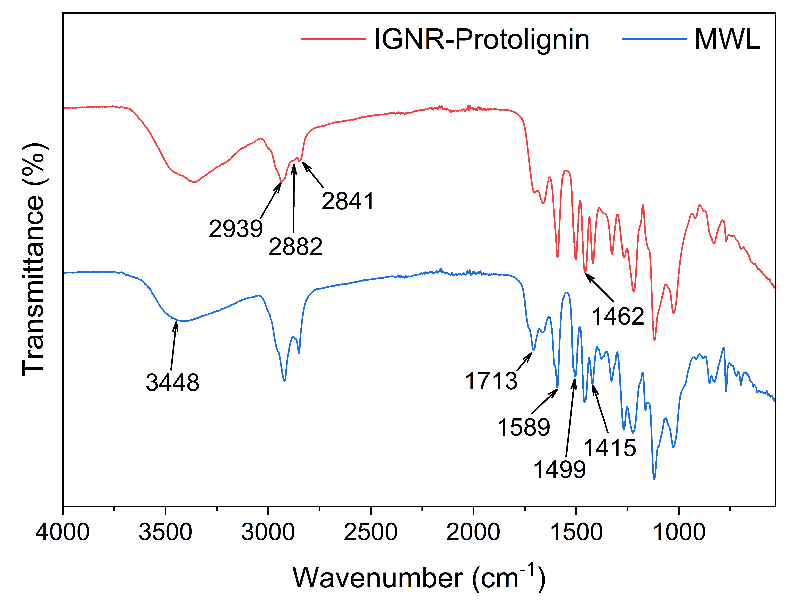


**Figure S6. FT-IR absorption spectra of IGNR-Protolignin and MWL**


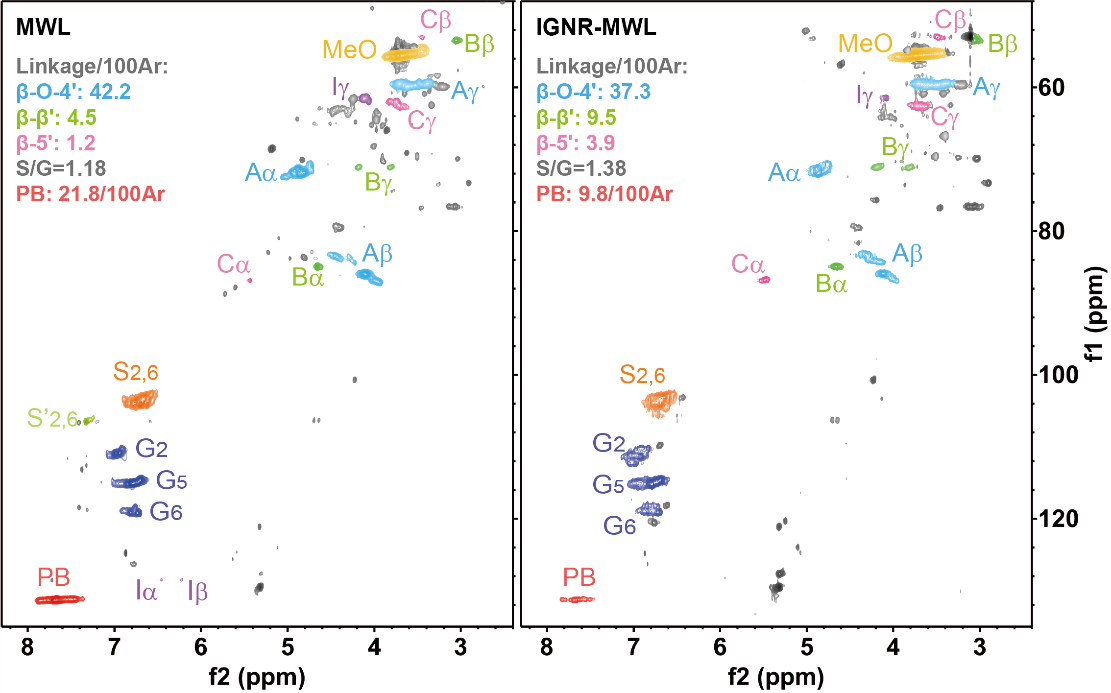


**Figure S7. 2D HSQC NMR of MWL and IGNR-MWL**


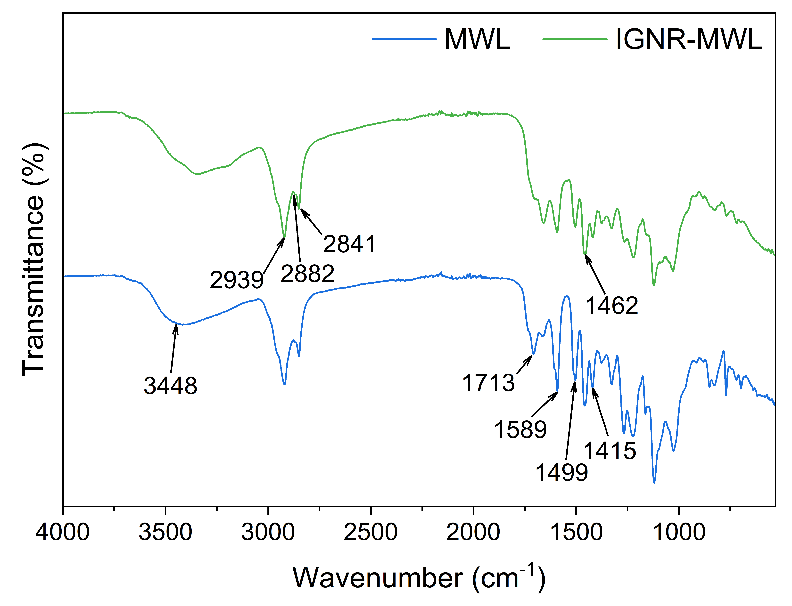


**Figure S8. FT-IR absorption spectra of MWL and IGNR-MWL**

**
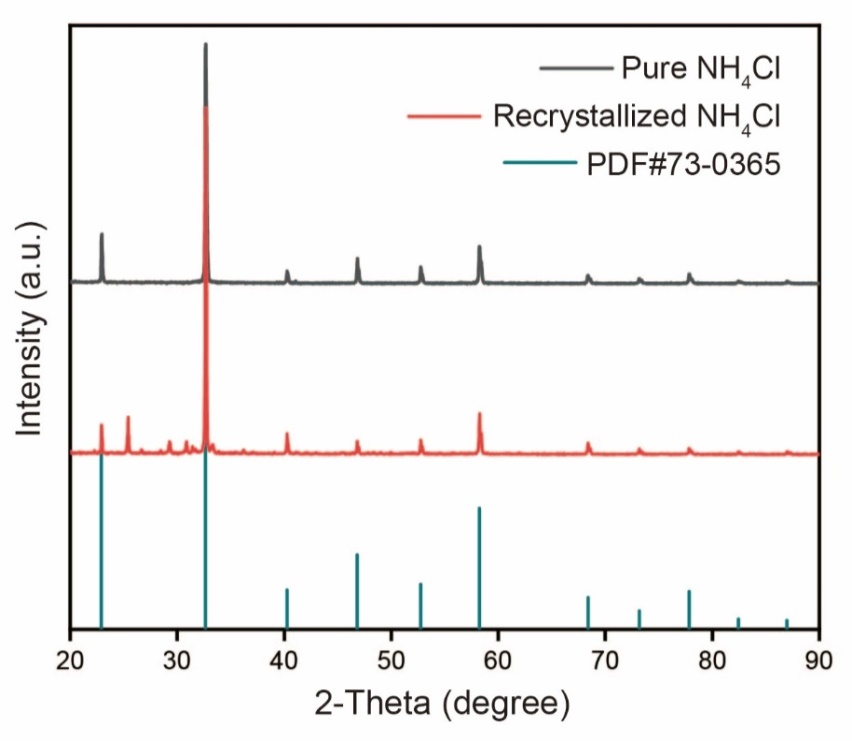
**

**Figure S9. XRD curves of recrystallized NH_4_Cl crystals**

**_
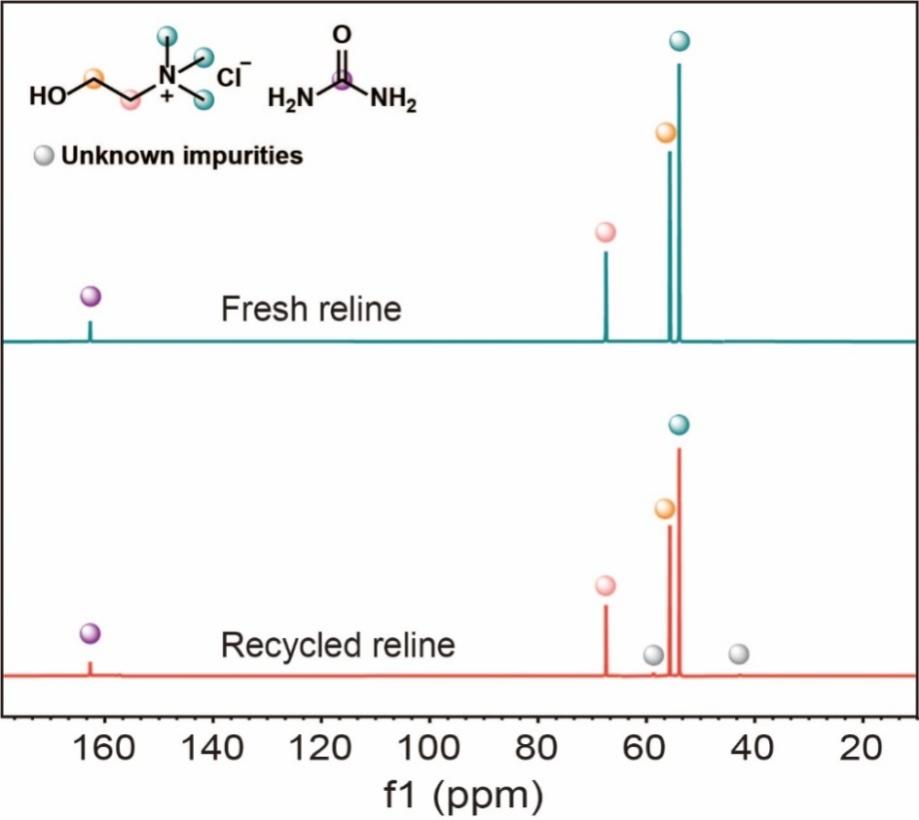
_**

**Figure S10.** **^13^C NMR spectra of fresh (unreacted) and recycled reline**

**
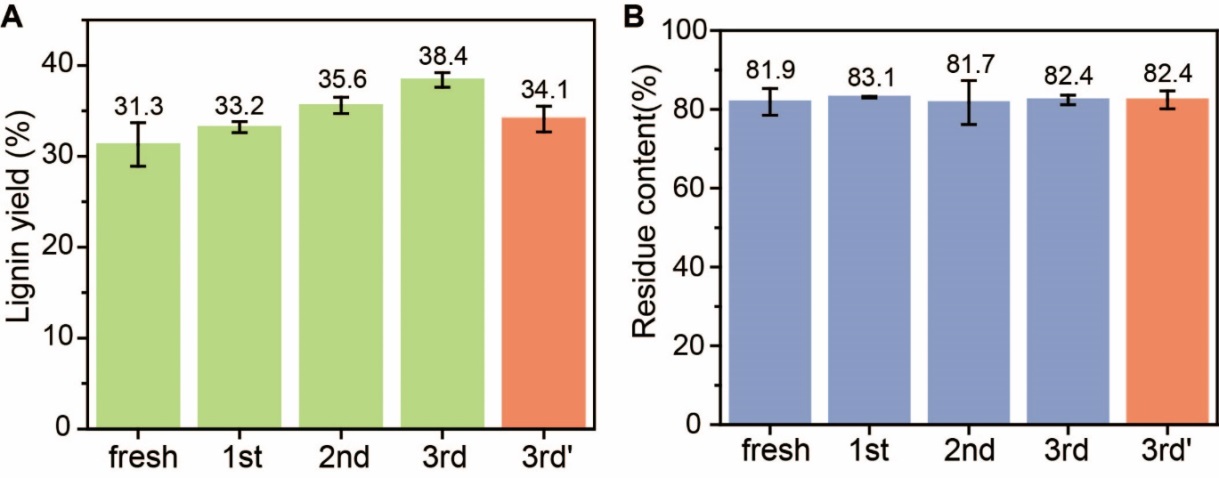
**

**Figure S11.** **Lignin yield (A) and residue content (B) in the IGNR system with recycled reline**

1st, 2nd, 3rd: extraction of protolignin by first, second and third recovery of reline; 3rd’: extraction of protolignin by adding 50% urea to third recovery of reline.

**
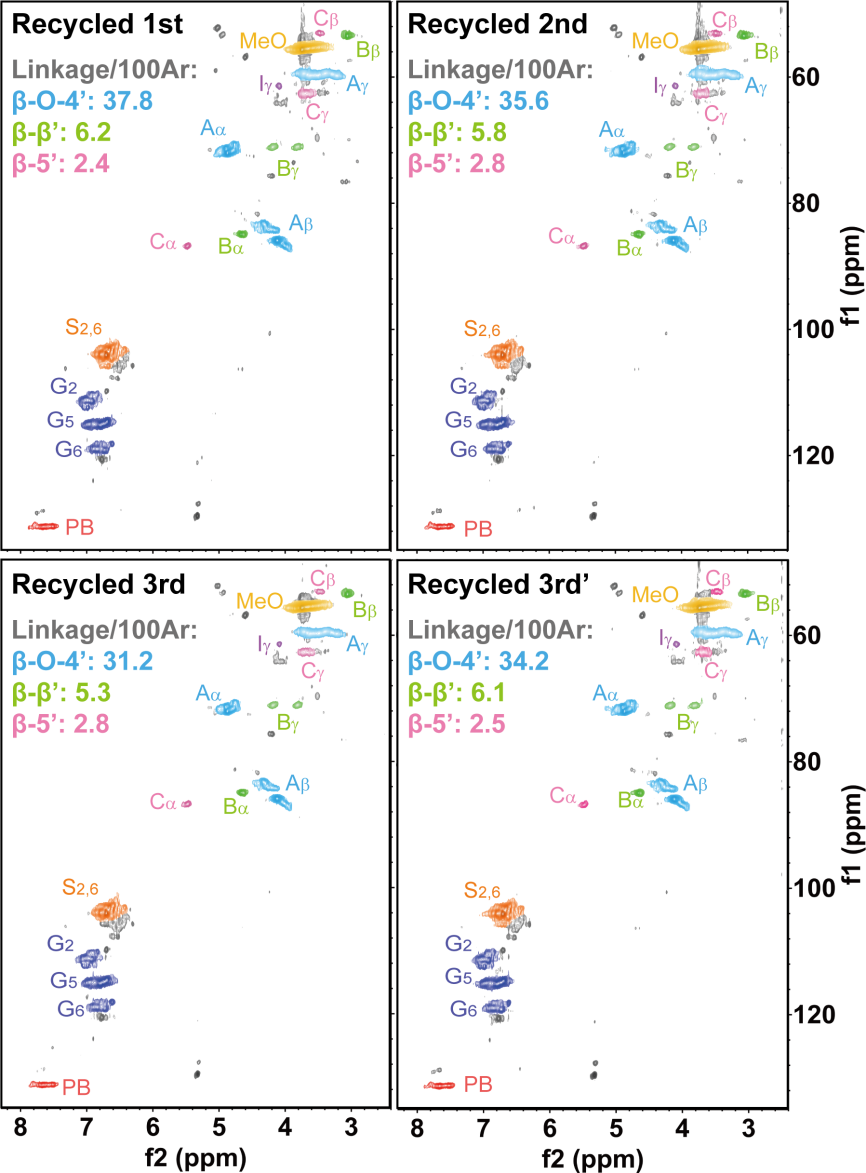
**

**Figure S12.** **2D HSQC-NMR of IGNR-Protolignin extracted by recycled reline**

**
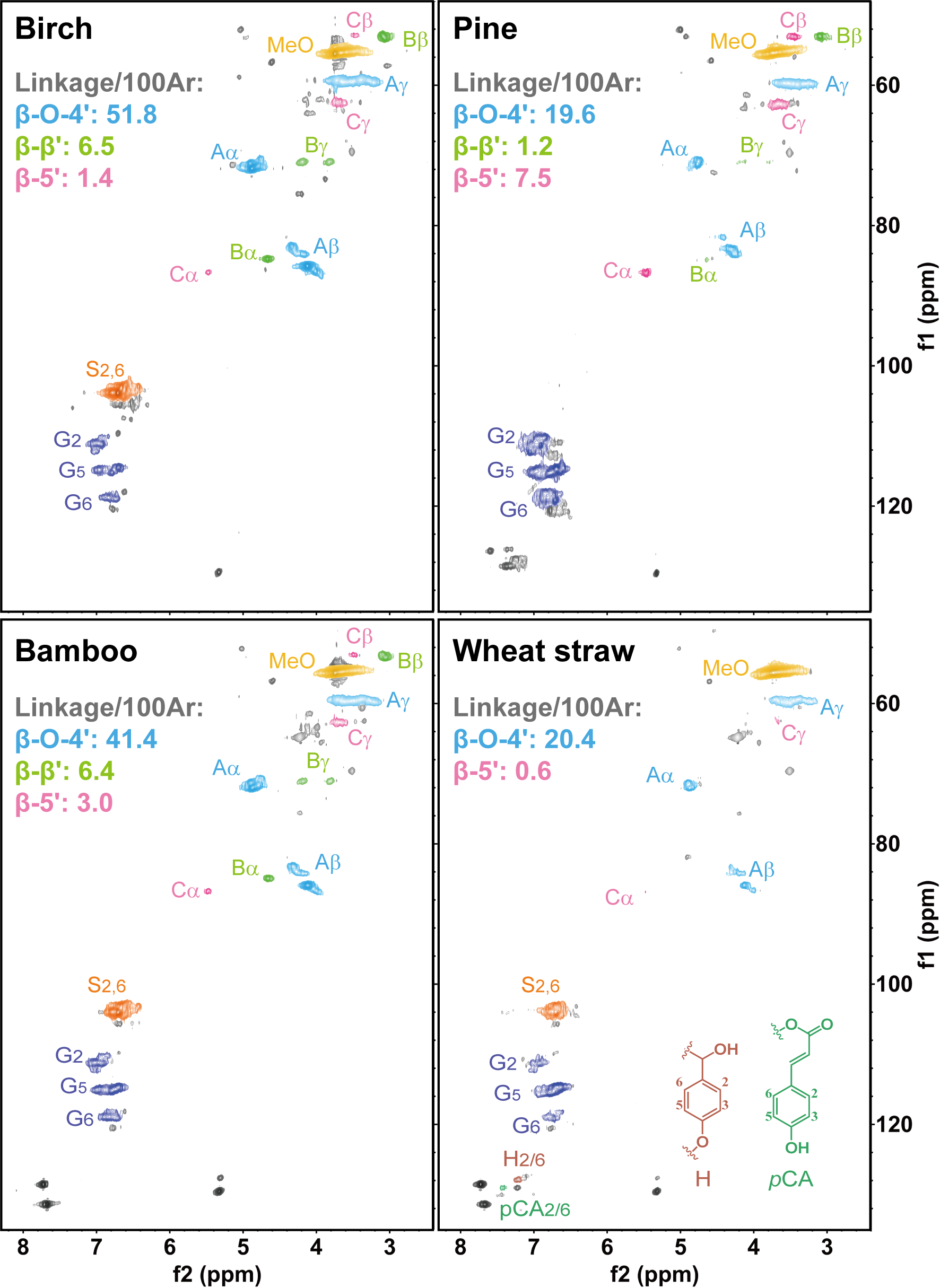
**

**Figure S13. 2D HSQC characterization of IGNR-Protolignin from different lignocelluloses**


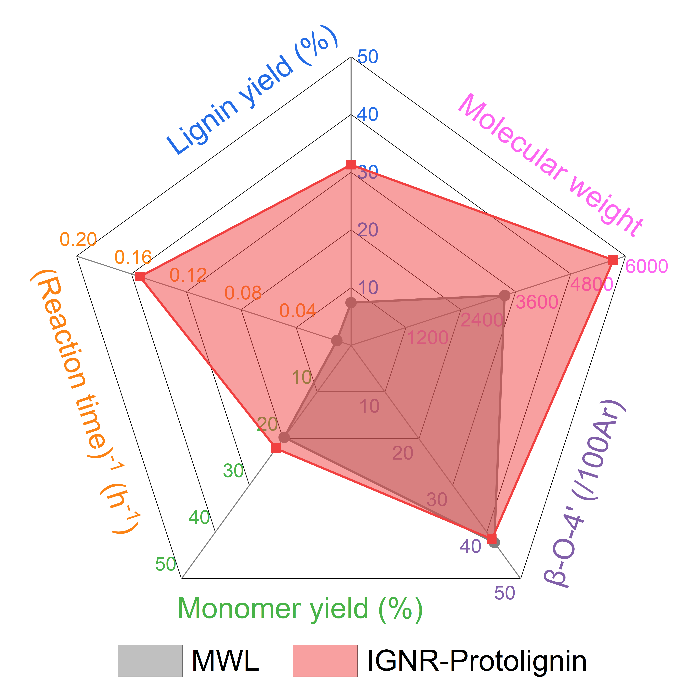


**Figure S14. Radar map comparison of MWL and IGNR methods.** The reaction time did not count the steps of lignin separation, precipitation and drying after the reaction was finished; the reaction time was 96h and 6.5h for MWL and IGNR-protolignin, respectively.

**Table S1.** **Composition analysis of the raw material and IGNR-Residue**

|  | Cellulose | Hemicellulose | Lignin |
| --- | --- | --- | --- |
| Raw material | 41.9±1.5% | 23.6±0.8% | 19.8±0.3% |
| IGNR-Residue | 40.0±1.3% | 22.9±1.0% | 13.2±0.7% |

**Table S2. Monosaccharide analysis of the raw material and IGNR-Residue**

|  | Glucose | Xylose | Mannose | Galacturonic acid | Glucuronic acid | Total sugar ratio |
| --- | --- | --- | --- | --- | --- | --- |
| Raw material | 54.2±0.20% | 39.5±0.60% | 5.1±0.20% | 0.9±0.01% | 0.3±0.04% | 100.0% |
| IGNR-Residue | 52.4±0.38% | 40.1±1.39% | 3.5±0.48% | 0.0±0.02% | 0.1±0.02% | 96.1±4.3% |

**Table S3. Relative molar abundance of the compounds identified in MWL in Figure S5**

| Label | Retention time | Compound | M_W_ (amu) | Relative abundance (%) | Origin |
| --- | --- | --- | --- | --- | --- |
| 1 | 9.541 | Phenol | 94 | 1.53 | H |
| 2 | 11.270 | 4-methoxyphenol | 124 | 1.40 | G |
| 3 | 13.017 | 2-methoxy-4-methylphenol | 138 | 3.57 | G |
| 4 | 14.244 | 4-ethyl-2-methoxyphenol | 152 | 0.73 | G |
| 5 | 14.740 | 2-methoxy-4-vinylphenol | 150 | 2.49 | G |
| 6 | 15.153 | 2,6-dimethoxyphenol | 154 | 3.71 | S |
| 7 | 15.748 | (Z)-2-methoxy-4-propenylphenol | 164 | 0.32 | G |
| 8 | 15.804 | Vanillin | 152 | 1.69 | G |
| 9 | 16.119 | 2,6-dimethoxy-4-methylphenol | 168 | 5.20 | S |
| 10 | 16.199 | 2-methoxy-4-propenylphenol | 164 | 2.72 | G |
| 11 | 16.781 | 4-ethyl-2,6-dimethoxyphenol | 182 | 0.92 | S |
| 12 | 16.897 | 2-propanone, 1-(4-hydroxy-3-methoxyphenyl)- | 180 | 0.41 | G |
| 13 | 17.027 | (Z)-4-(butenyl)guaiacol | 178 | 0.27 | G |
| 14 | 17.147 | 2,6-dimethoxy-4-vinylphenol | 180 | 3.41 | S |
| 15 | 17.399 | 4-allyl-2,6-dimethoxyphenol | 194 | 3.21 | S |
| 16 | 17.440 | Homosyringaldehyde | 196 | 2.77 | S |
| 17 | 17.779 | 4-propenyl-2,6-dimethoxyphenol | 194 | 0.93 | S |
| 18 | 17.947 | Syringaldehyde | 182 | 2.85 | S |
| 19 | 18.172 | 4-allyl-2,6-dimethoxyphenol | 194 | 3.47 | S |
| 20 | 18.427 | Acetosyringone | 196 | 1.32 | S |
| 21 | 18.563 | Coniferyl alcohol | 180 | 2.76 | G |
| 22 | 18.982 | Acetosyringone | 196 | 0.49 | S |
| 23 | 19.603 | (Z)-Sinapyl alcohol | 210 | 0.20 | S |
| 24 | 20.009 | Sinapaldehyde | 208 | 0.08 | S |
| 25 | 20.070 | (Z)-Sinapyl alcohol | 210 | 0.21 | S |

**Table S4. Relative molar abundance of the compounds identified in IGNR-Protolignin in Figure S5**

| Label | Retention time | Compound | M_W_ (amu) | Relative abundance (%) | Origin |
| --- | --- | --- | --- | --- | --- |
| 1’ | 9.506 | Phenol | 94 | 1.56 | H |
| 2’ | 11.222 | Guaiacol | 124 | 1.95 | G |
| 3’ | 12.785 | 2-methoxy-4-methylphenol | 138 | 0.06 | G |
| 4’ | 13.015 | 2-methoxy-4-methylphenol | 138 | 1.54 | G |
| 5’ | 14.743 | 2-methoxy-4-vinylphenol | 150 | 1.99 | G |
| 6’ | 15.172 | 2,6-dimethoxyphenol | 154 | 6.23 | S |
| 7’ | 15.307 | 2-methoxy-4-propylphenol | 166 | 0.26 | G |
| 8’ | 15.752 | 2-methoxy-4-propenylphenol | 164 | 0.29 | G |
| 9’ | 15.809 | Vanillin | 152 | 1.17 | G |
| 10’ | 16.123 | 2,6-dimethoxy-4-methylphenol | 168 | 3.51 | S |
| 11’ | 16.201 | 2-methoxy-4-propenylphenol | 164 | 2.26 | G |
| 12’ | 16.783 | 4-ethyl-2,6-dimethoxyphenol | 182 | 0.79 | S |
| 13’ | 16.913 | 2-propanone, 1-(4-hydroxy-3-methoxyphenyl)- | 180 | 0.62 | G |
| 14’ | 17.027 | (Z)-4-(butenyl)guaiacol | 178 | 0.16 | G |
| 15’ | 17.162 | 2,6-dimethoxy-4-vinylphenol | 180 | 3.87 | S |
| 16’ | 17.403 | 4-allyl-2,6-dimethoxyphenol | 194 | 2.05 | S |
| 17’ | 17.781 | 4-propenyl-2,6-dimethoxyphenol | 194 | 1.29 | S |
| 18’ | 17.977 | Syringaldehyde | 182 | 4.42 | S |
| 19’ | 18.086 | Coniferyl alcohol | 180 | 2.02 | G |
| 20’ | 18.440 | Acetosyringone | 196 | 2.50 | S |
| 21’ | 18.597 | Coniferyl alcohol | 180 | 6.90 | G |
| 22’ | 18.647 | Syringylacetone | 224 | 1.74 | S |
| 23’ | 19.001 | 1-propanone, 1-(4-hydroxy-3,5-dimethoxyphenyl)- | 210 | 0.77 | S |
| 24’ | 19.591 | Sinapyl alcohol | 210 | 2.03 | S |
| 25’ | 20.092 | Sinapyl alcohol | 210 | 5.66 | S |

**Table S5. Molecular weight determination of MWL and IGNR-Protolignin by GPC**

| **Lignin** | ***M*_n_ (g/mol)** | ***M*_w_ (g/mol)** | **PDI** |
| --- | --- | --- | --- |
| MWL | 1048 | 3358 | 3.2 |
| IGNR-Protolignin | 1403 | 5735 | 4.1 |

**Table S6. Comparison of MWL and IGNR methods**

|  | **MWL method** | **IGNR method** |
| --- | --- | --- |
| **Structures** | Structures are both close to protolignin and have similar activity | |
| **Molecular weight** | Smaller molecular weight but uniform distribution | Large molecular weight and wide distribution |
| **Yield** | 7.4wt% | 31.3wt% |
| **Reaction time** | >96h (Ball milling 48h + Solvent extraction 48h) | <12h (Reline Synthesis 0.5h + Reaction 6h) |
| **Reaction Scale** | Small amount and need to remove the residual sugar | Mass production and solvent recovery |
| **Environmental issues** | Dioxane is highly toxic and volatile; hydrochloric acid is a strong acid with strong volatility | Reline is green, non-toxic and recyclable; acetone is toxic but recyclable |

**Table S7. Comparison of protolignin obtained by different extraction methods**

| **Lignocellulose** | **Solvents or enzymes** | **Time**^a^  **(h)** | **β-O-4' content (/100Ar)** | **Lignin** | **Ref.** |
| --- | --- | --- | --- | --- | --- |
| Poplar | Reline (ChCl/Urea) | 6 | 53.9 (41.5)^b^ (/S+G+PB) | IGNR-Protolignin | This work |
| Poplar | Cellic CTec2^c^, 96% dioxane | 122 | 61 (/S+G) | CEL^d^ | Ref 8 |
| Poplar^e^ | Cellic CTec2 | 103 | 60 (/S+G_2_) | DEL | Ref 9 |
| Poplar^f^ | Cellulase, β-glucosidase, 96% dioxane | 74 | 55.4 (/S+G) | CEL | Ref 10 |
| Poplar^g^ | Cellic CTec 2, Cellic HTec 2^c^, streptomyces griseus protease | 110 | 61.1 (/S+G) | CEL | Ref 11 |
| Poplar^h^ | 96% dioxane, Cellic CTec2 | 120 | 63.5 (/S+G_2_) | CEL | Ref 12 |
|  | 80% dioxane, Cellic CTec2 |  | 64 (/S+G_2_) |  |  |
| Eucalyptus | Toluene, Onozuka SS1500^c^ | 149 | 55.3 | CEL | Ref 13 |
|  | NaOH (aq), cellulase, β-glucosidase | 77 | 53.6 | SREL |  |
| Eucalyptus | 80% dioxane, 95% ethanol | 17 | 45.2 (/S+G_2_) | MWL | Ref 14 |
|  | 80% dioxane, Cellic CTec2 | 65 | 58.1 (/S+G_2_) | CEL |  |
|  | 80% dioxane, Cellic CTec2 | 113 | 63.8 (/S+G_2_) | EHL |  |
|  | Cellic CTec2 | 103 | 60.8 (/S+G_2_) | DEL |  |
| Loblolly pine | Toluene, 96% dioxane | ＞24 | 41 | MWL | Ref 15 |
|  | Toluene, Onozuka SS1500 | ＞192 | 47 | CEL |  |
| Wheat straw steam | Cellulase, β-glucosidase, xylanase 96% dioxane, 90% acetic acid | 122-128 | 64 (/S+G+H) | CEL | Ref 16 |
| Wheat straw leaf |  |  | 56 (/S+G+H) |  |  |

^a^Only the time for critical solvent or enzyme treatment was included here, not other minor steps such as filtration, concentration, drying, etc. ^b^The number (41.5) was divided by a factor of a 1.3 to better reflect the actual amount of β-O-4' units per 100 aromatic units.^6^ However, there is no coefficient optimization in the comparative literature, so we also showed the β-O-4' content before optimization. ^c^Cellic CTec2 and Onozuka SS1500 are cellulases; Cellic HTec 2 is a hemicellulase. ^d^CEL, DEL, SREL, MWL, EHL represent cellulolytic enzyme lignin, double enzymatic lignin, swollen residual enzyme lignin, milled wood lignin, and enzymatic hydrolysis lignin respectively. ^e-h^Specific poplar species are ^e^populus tomentosa, ^f^populus trichocarpa, ^g^Populus trichocarpa × deltoides, and ^h^triploid of Populus tomentosa Carr, respectively.

**Reference**

1. Dawange, M., Galkin, M.V., and Samec, J.S.M. (2015). Selective aerobic benzylic alcohol oxidation of lignin model compounds: route to aryl ketones. ChemCatChem *7*, 401-404.
2. Liu, Y., Deak, N., Wang, Z., Yu, H., Hameleers, L., Jurak, E., Deuss, P.J., and Barta, K. (2021). Tunable and functional deep eutectic solvents for lignocellulose valorization. Nat. Commun. *12*, 5424.
3. Hou-Min, C., Ellis, B.C., and Wynford, B. (2009). Comparative studies on cellulolytic enzyme lignin and milled wood lignin of sweetgum and spruce. Holzforschung *29*, 153-159.
4. Foster, C.E., Martin, T.M., and Pauly, M. (2010). Comprehensive compositional analysis of plant cell walls (lignocellulosic biomass) Part I: Lignin. J. Vis. Exp. *37*, e1745.
5. Robinson, A.R., and Mansfield, S.D. (2009). Rapid analysis of poplar lignin monomer composition by a streamlined thioacidolysis procedure and near-infrared reflectance-based prediction modeling. Plant J. *58*, 706-714.
6. Zijlstra, D.S., Lahive, C.W., Analbers, C.A., Figueirêdo, M.B., Wang, Z., Lancefield, C.S., and Deuss, P.J. (2020). Mild organosolv lignin extraction with alcohols: the importance of benzylic alkoxylation. ACS Sustain. Chem. Eng. *8*, 5119-5131.
7. Ren, T., You, S., Zhang, Z., Wang, Y., Qi, W., Su, R. and He, Z. (2021). Highly selective reductive catalytic fractionation at atmospheric pressure without hydrogen. Green Chem. *23*, 1648-1657.
8. He, D., Zhuang, J., Jiang, Y., Xie, D., Yoo, C.G., and Yang, Q. (2021). Fractionation of poplar wood using a bifunctional aromatic acid under mild conditions. ACS Sustain. Chem. Eng. *9*, 5364-5376.
9. Wang, H., Wang, B., Wen, J., Yuan, T., and Sun, R. (2017). Structural characteristics of lignin macromolecules from different eucalyptus species. ACS Sustain. Chem. Eng. *5*, 11618-11627.
10. Li, M., Cao, S., Meng, X., Studer, M., Wyman, C.E., Ragauskas, A.J., and Pu, Y. (2017). The effect of liquid hot water pretreatment on the chemical-structural alteration and the reduced recalcitrance in poplar. Biotechnol. Biofuels *10*, 237.
11. Meng, X., Parikh, A., Seemala, B., Kumar, R., Pu, Y., Christopher, P., Wyman, C.E., Cai, C.M., and Ragauskas, A.J. (2018). Chemical transformations of poplar lignin during cosolvent enhanced lignocellulosic fractionation process. ACS Sustain. Chem. Eng. *6*, 8711-8718.
12. Chen, T., Wang, B., Shen, X., Li, H., Wu, Y., Wen, J., Liu, Q., and Sun, R. (2017). Assessment of structural characteristics of regenerated cellulolytic enzyme lignin based on a mild DMSO/[Emim]OAc dissolution system from triploid of *Populus tomentosa* Carr. RSC Adv. *7*, 3376-3387.
13. Wen, J., Sun, S., Yuan, T., and Sun, R. (2015). Structural elucidation of whole lignin from Eucalyptus based on preswelling and enzymatic hydrolysis. Green Chem. *17*, 1589-1596.
14. Wang, H., Ma, C., Li, H., Chen, T., Wen, J., Cao, X., Wang, X., Yuan, T., and Sun, R. (2020). Structural variations of lignin macromolecules from early growth stages of poplar cell walls. ACS Sustain. Chem. Eng. *8*, 1813-1822.
15. Holtman, K.M., Chang, H., and Kadla, J.F. (2004). Solution-state nuclear magnetic resonance study of the similarities between milled wood lignin and cellulolytic enzyme lignin. J. Agr. Food Chem. *52*, 720-726.
16. Jiang, B., Cao, T., Gu, F., Wu, W., and Jin, Y. (2017). Comparison of the structural characteristics of cellulolytic enzyme lignin preparations isolated from wheat straw stem and leaf. ACS Sustain. Chem. Eng. *5*, 342-349.
